# Manual therapy in the treatment of carpal tunnel syndrome in diabetic patients: A randomized clinical trial

**DOI:** 10.22088/cjim.9.3.283

**Published:** 2018

**Authors:** Ghadam Ali Talebi, Payam Saadat, Yahya Javadian, Mohammad Taghipour

**Affiliations:** 1 Mobility Impairment Research Center, Health Research Institute, Babol University of Medical Sciences, Iran; 2 Clinical Research Development Unit of Ayatollah Rouhani Hospital, Babol University of Medical Sciences, Babol, Iran; 3 3.Social Determinants of Health Research Center, Health Research Institute, Babol University of Medical Sciences, Babol, Iran

**Keywords:** Diabetic Patient, Carpal tunnel syndrome, Manual Therapy

## Abstract

**Background:**

Generally, conservative interventions including physiotherapy modalities and manual therapy have been recommended in the management of carpal tunnel syndrome (CTS), but this subject has not been studied in diabetic patients with CTS. Therefore the aim of this study was to investigate the effects of manual therapy on diabetic patients with CTS.

**Methods:**

Thirty diabetic patients with CTS were randomly divided into two equal groups: modality group and manual therapy group. Participants in the modality group received transcutaneous electrical nerve stimulation (TENS) and therapeutic ultrasound (US) and patients in the manual therapy group received manual techniques for the median nerve and its surrounding structures. Interventions were applied 3 times weekly for 4 weeks in both groups. Visual analogue scale (VAS), symptom severity scale (SSS), functional status scale (FSS) and median neurodynamic test (MNT) were evaluated before and after the interventions in both groups. Paired t-test and independent t-test were used for statistical analysis.

**Results:**

Paired t-test revealed that all of the outcome measures had a significant change in the manual therapy group, whereas only the VAS and SSS changed significantly in the modality group at the end of 4 weeks. Independent t-test showed that the variables of SSS, FSS and MNT in the manual therapy group improved significantly greater than the modality group.

**Conclusions:**

Manual therapy techniques applied to mechanical interface of the median nerve and nerve mobilization possess more appropriate and valuable effects on hand difficulties than modalities in diabetic patients with CTS.

Carpal tunnel syndrome (CTS) is the most common entrapment neuropathy in upper limbs which is caused by entrapment and compression of the median nerve at wrist within the carpal tunnel. In many cases, overuse / repetitive trauma and prolonged incorrect position of the hand or wrist during the occupational activities are the main causes of CTS (1). Although most causes are idiopathic, CTS may be associated with some systemic conditions such as diabetes mellitus. CTS is the most common entrapment neuropathy in patients with diabetes, which may be due to metabolic changes, repeated undetected trauma, accumulation of fluid or edema within the carpal tunnel and/or diabetic cheiroarthropathy (2-4). Diabetic patients are more prone to entrapment in anatomically constrained channels since the peripheral nerves indicate both functional impairment and structural changes because of abnormal glucose metabolism and consequent metabolic alterations. (2). In general, conservative treatment is recommended for mild to moderate CTS.

Splinting and physiotherapy modalities such as ultrasound (US) and electrotherapy are proposed for management of CTS (5-9). In entrapment neuropathy like CTS, the gliding of the peripheral nerve and its capability to sustain tension are partially limited (10). In addition, adhesion, fibrosis and possibly scar tissue may occur around the median nerve within the carpal tunnel, which causes pathomechanic and pathophysiologic consequences for the nerve (11). It seems that changes in neural adaptation and excursion of the median nerve in CTS may result in reproduction or increment of hand symptoms and or abnormal response to neurodynamic testing (10-13). There is a relationship between the pathomechanical impairment of the nerve and pathophysiological process of the nerve that must be considered in treatment planning (12). Manual therapy techniques include soft tissue and carpal bone mobilization (14-17) and also median nerve mobilization will potentially reduce the pressure existing around the nerve and improve blood flow of the nerve,which help nerve heal and improve CTS symptoms (18,19). To the best of our knowledge, no previous studies investigated the effects of the manual therapy or physiotherapy modalities in diabetic populations with CTS. In fact, the presence of diabetes was a rule-out criterion in all of the previous studies. Therefore, the aim of the current study was to investigate the effects of the manual therapy (emphasized on nerve mobilization) and compare with those of physiotherapy modalities (ultrasound and TENS) on hand symptoms and neurodynamics of median nerve in diabetic patients with CTS. 

## Methods

**Figure 1:**
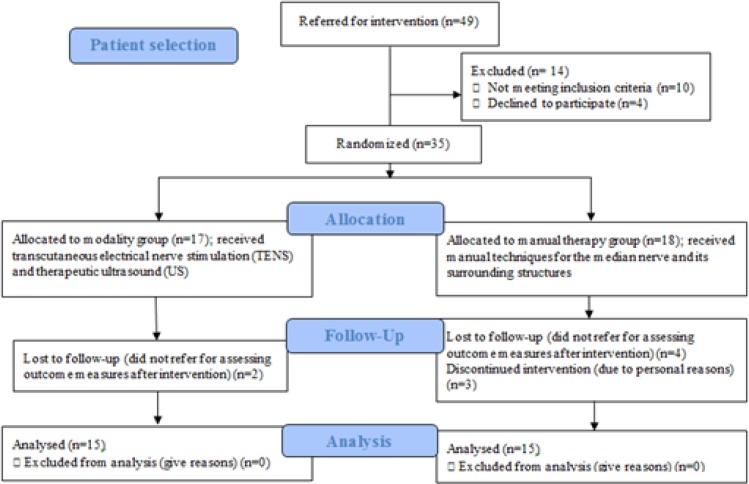
Patient Selection

**Participants: **Sample size was calculated according to VAS variable from our previous study by considering α= 0.05 and β=0.2(19). Totally, thirty diabetic patients with CTS aged 30–65 years, referring to Ayatollah Rouhani and Amirkola Hospitals, participated in this randomized clinical trial. Randomization was performed through simple random method (fig 1). Also, the staff assessing the outcome measures and analyzing the data were also blinded to the group allocations. The inclusion criteria were: a) patients with CTS diagnosed by a neurologist b) patients with the complaint of pain and paresthesia in the distribution of median nerve within the hand for at least 6 months c) patients with positive tinel sign, positive phalen sign, and d) patients with diabetes at least 2 years. Exclusion criteria included patients with the history of carpal tunnel release, previous steroid injection, cervical radiculopathy, metabolic disorders other than diabetes, pregnancy, history of neck / shoulder or arm trauma and atrophy of thenar muscles. Written informed consent form was filled out by all subjects and the protocol was approved by Babol University of Medical Sciences Ethics Committee (code no: MUBABOL.REC.1394.103). 

This study has been registered at Iranian Registry of Clinical Trials (IRCT) with registration number 201508182851N4. The patients were randomly assigned into two groups: modality group (15 patients) and manual therapy group (15 patients). Randomization has been done by a simple random allocation. Procedure included assignment of alternating patients sequentially to each group. So, the patients with even number were assigned to modality group and patients with odd number to manual therapy group (20). The participants were blinded for both grouping and treatment method. The examiner who collected primary and secondary outcome measures before and after treatment procedures while the data analyst was unaware of the assigned treatment (21).

**Figure 2:**
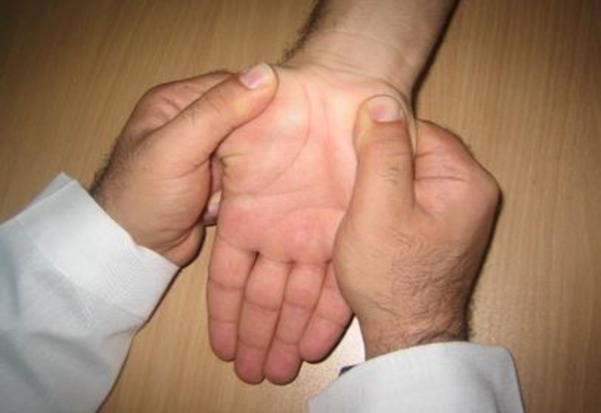
Transverse carpal ligament release

**Figure 3:**
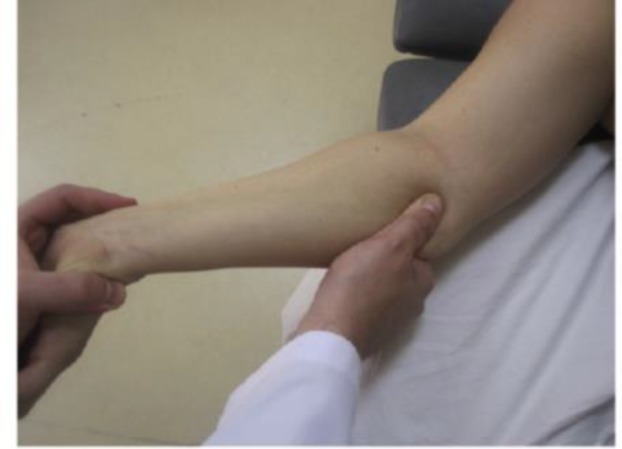
Soft tissue manipulation of the pronator teres

**Figure 4:**
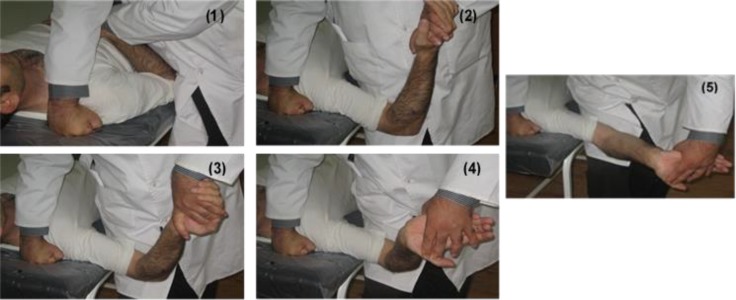
Stages of median nerve neurodynamic testing. These maneuvers with some modifications can be used for nerve mobilization by gliding and tension techniques according to assessment findings.

**Intervention: **Participants in the modality group received transcutaneous electrical nerve stimulation (TENS) and therapeutic ultrasound. Patients in the manual therapy group received the combination of manual techniques for mechanical interfaces around the median nerve and neuromobilization. Interventions were used 3 times weekly for 4 weeks in both groups. TENS (frequency of 80 Hz, pulse duration of 60 µs) at the level of comfortable tingling sensation was applied for 20 minutes each session. Therapeutic US (frequency of 1 MHz and intensity of 1 W/cm^2^) was applied for 5 minutes per session on the palmar surface of the carpal tunnel (19). Manual techniques consisted of carpal bone mobilization, transverse carpal ligament release (fig 2), palmar fascia release of the hand, soft tissue manipulation in the distal arm and proximal forearm areas (14-17) (fig 3) and median nerve mobilization techniques (fig 4). After carpal bones and soft tissue mobilization, median nerve mobilization was applied and progressed slowly and carefully based on Shacklocks approach (23). Manual techniques were collectively administered 25 minutes for each session. 

**Primary Outcome measures:**

**Self-report hand pain and discomfort: **A visual analogue scale (VAS) via 11-point numerical pain rating scale (0=no pain to 10=maximum pain) was used to assess the current level of pain and hand discomfort (19).

**The Boston Questionnaire: **The questionnaire comprises two parts, namely the Symptom Severity Scale (SSS) and the Functional Status Scale (FSS). In the SSS, there are 11 questions; responses may be scored one (mildest) point to five (most severe) points. The overall result is the calculated mean of all 11 scores. In the FSS, there are eight questions assessing the difficulty in performing selected activities. The overall score for functional status is calculated as the mean of all eight questions. Thus, a higher symptom severity or functional status score indicates worse symptoms or dysfunction (22). The Boston Questionnaire is a standardized, patient-based outcome measure of symptom severity and functional status in patients with carpal tunnel syndrome (23). The validity and reliability of the Persian version of Boston Questionnaire have been approved by several studies (24, 25). 

**Secondry Outcome measures:**

**Neurodynamics: **Median neurodynamic test (MNT) consists of a series of passive movements applied to the upper extremity to identify neural tissue dynamics (12, 26). Research findings have shown that the MNT is a highly reliable tool for assessment in CTS patients (25). MNT was performed with the following standardized sequence (26, 5) 1) the shoulder girdle was slightly depressed downward, 2) the arm was abducted slightly more than 90 degrees, 3) the forearm was fully supinated and the shoulder externally rotated, 4) the fingers were extended, and 5) finally the elbow slowly was extended (Fig 3). 

At the time of hand symptom reproduction, the test was stopped and the elbow extension angle was then measured. The MNT was repeated three times, with 2-min rest interval and the average of measurements was used for analysis.

**Data collection and analysis: **Descriptive statistics are given as mean±SD. Based on the results of Kolmogorov-Smirnov test, the variables had normal distribution, so parametric tests were used for data analysis. Within the groups, comparisons were carried out by paired t-test and comparisons between the groups were performed using independent t-test. A p<0.05 was considered statistically significant. Data were analyzed using SPSS Version 24.

## Results

The mean values of VAS, SSS and FSS were 6.58, 29.91 and 16.5 in modality group, respectively; while these values were 7.08, 29.91 and 18.33 in manual therapy group respectively. Demographic and clinical characteristics of all patients at baseline are demonstrated in table 1. There was no significant difference between the two groups regarding age, duration of hand symptoms, duration of diabetes, MNT, VAS, SSS, and FSS at baseline. Paired t-test revealed a significant change in the mean values of VAS (p=0.001) and SSS (p=0.001), in the modality group, but no significant change in FSS (p=0.24) and MNT (p=0.22) at the end of 4^th^ weeks (table 2). Significant improvement was found in all of outcome measures (VAS, SSS, FSS, and MNT) in the manual therapy group (table 2).

**Table1:** Patient’s characteristics at baseline

**Group **	**Modality** **(N=15)**	**Manual therapy** **(N=15)**
Age (years)	50.17±10.16	49±10.18
Duration of hand symptoms (Month)	28.66±24.57	32.25±31.21
Duration of diabetes (years)	3.33±1.07	3.66±1.49
MNT* (Angle of elbow)	47.33±5.74	49.25±7.37
VAS** (cm)	6.58±1.37	7.08±1.56
SSS***	29.91±7.24	29.91±9.65
FSS****	16.5±6.20	18.33±8.31

* MNT: median neurodynamic test

** VAS: visual analogue scale

*** SSS: symptom severity scale

**** FSS: functional status scale

Independent t-test showed that the variables of SSS, FSS and MNT in the manual therapy group improved significantly more than the modality group (p<0.05) (table 3).

**Table 2:** Results of paired t-test in comparing Variables before and after the intervention within the groups

	**Modality**			**Manual Therapy**		
	**Before** **Mean±SD**	**After** **Mean±SD**	**P value**	**Before** **Mean±SD**	**After** **Mean±SD**	**P value**
Vas*	6.58±1.37	4.41±1.31	0.000	7.08±1.56	3.75±2.22	0.000
SSS**	29.91±7.24	25.41±6.25	0.000	29.91±9.65	19.25±6.25	0.000
FSS***	16.5±6.20	15.75±5.31	0.241	18.33±8.31	14.33±6.25	0.008
MNT****	47.33±5.74	46.00±5.20	0.223	49.25±7.34	34.25±6.53	0.000

* VAS: visual analogue scale

** SSS: symptom severity scale

*** FSS: functional status scale

**** MNT: median neurodynamic test

**Table 3:** Results of independent t-test in comparing of improvement (%) between the two groups at the end of 4^th^ weeks

	**Modality** **Mean±SD**	**Manual Therapy** **Mean±SD**	**P value**
VAS* (cm)	32.29±16.08	47.03±25.81	0.141
SSS**	15.04±6.83	35.64±16.92	0.006
FSS***	4.54±4.33	21.18±14.55	0.043
MNT****	2.81±1.17	30.45±9.42	0.000

* VAS: visual analogue scale

** SSS: symptom severity scale

*** FSS: functional status scale

**** MNT: median neurodynamic test

## Discussion

The results of the present study indicated that using 4-week physiotherapy modalities (TENS and therapeutic ultrasound) may be helpful to improve the subjective problems (VAS and SSS) without beneficial effects on hand functional status (FSS) and median neurodynamics (MNT). All outcome measures in diabetics with CTS who received manual therapy techniques improved after 4 weeks. Additionally, to compare the variables between two groups at the end of 4^th ^weeks revealed significant improvement for all outcome measures except VAS in the manual therapy group than modality group (table 3).

Some previous studies reported that the physiotherapy modalities have beneficial effects on pain relief and sensory symptoms in patients with CTS (5-9). It is to be noted that the subjects participating in mentioned studies were not diabetics. As we know, the nature of nerve pathology in CTS is somewhat different in diabetics than CTS patients with only simple mechanical nerve entrapment (2, 3). Based on our findings, it appears that TENS and therapeutic ultrasound possess limited effects on hand symptoms without useful effects on functional abilities of the hand and median nerve mobility in diabetic patients with CTS. Definitely, this issue may be affected by the severity and duration of diabetes and CTS, which needs more precise studies in the future. Some studies reported that the manual therapy techniques including soft tissue and carpal bone mobilization (14-17) and median nerve mobilization are useful to improve the CTS symptoms (18, 19). They postulated that these techniques potentially reduce the pressure existing around the nerve, improve the blood flow of the nerve and prevent the adherence of the nerve to surrounding tissues. Diabetes disease leads to vascular dysfunction, reduced nerve blood flow and endoneurial hypoxia (3), therefore the observed improvements in hand symptoms (FSS), functional capabilities of the hand (FSS) and median nerve neurodynamics (MNT) in the manual therapy group compared to modality group may be attributed to the potential effects of manual therapy on reducing the swelling around the nerve, increasing the blood flow of the nerve and improving neurodynamics, just as implicated in some sources (12, 26). 

Findings of the current study, similar to above mentioned reports, indicated that the manual therapy techniques focused on soft tissue / carpal bone mobilization and median nerve mobilization had useful effects on hand difficulties in patients with CTS and diabetes. With regard to the mechanism of the effectiveness of the manual therapy, Shocklock has expressed that there is a relationship between the pathophysiological (which is seen in diabetes) and pathomechanical (such as swelling around the nerve, adherence of nerve to surrounded tissues and disturbance in nerve mobility) processes of the nerve (26). Hence, diabetes increases the possibility of the mechanical problems of the nerve. On the other hand, it seems that the manual therapy included soft tissue and carpal bone mobilization as well as neuromobilization techniques improve the mechanical function of the nerve and consequently possess helpful effects on physiological disturbance of the nerve. Using large sample size, following-up the patients and analysis based on severity of the diabetes disease and CTS should be considered in the future study. Additionally, the use of Electrophysiological evaluation (EMG and NCV) recommended bettering explanation of clinical findings in future study.

In conclusion Physiotherapy modalities (TENS and ultrasound) have little useful effects on hand sensory discomfort in diabetic patients with CTS, but the manual therapy techniques applied to mechanical interface of the median nerve and nerve mobilization possess appropriate and valuable effects on hand difficulties in these patients. 
